# Dermatofibrosarcoma protuberans of the breast: A case report

**DOI:** 10.3892/ol.2014.2291

**Published:** 2014-06-26

**Authors:** JIN-QUN JIANG, ZHAN HUANG, LIN-HUI WANG, SAN-DI SHEN, HAI LU

**Affiliations:** 1 Department of Clinical Laboratory, Yuebei People’s Hospital, Shaoguan, Guangdong 512026, P.R. China; 2 Department of Breast Surgery, Yuebei People’s Hospital, Shaoguan, Guangdong 512026, P.R. China; 3 Department of Pathology, Yuebei People’s Hospital, Shaoguan, Guangdong 512026, P.R. China

**Keywords:** breast, dermatofibrosarcoma protuberans

## Abstract

Dermatofibrosarcoma protuberans (DFSP) is a rare, metastasizing tumor of the deep dermis and subcutaneous tissue. While it frequently occurs in the trunk and extremities, breast involvement has rarely been reported. In the present case, imaging and pathological technologies were used to detect DFSP of the breast. Surgical excision with wide margins (>3 cm) and pathology revealed spindle cells arranged in storiform patterns and short fascicles which were crucially CD34-positive, enabling a definitive diagnosis prior to surgery.

## Introduction

Dermatofibrosarcoma protuberans (DFSP) is an uncommon, slow-growing, low-grade sarcoma of putative dermal fibroblastic origin, all recurrence is *in situ* and rarely metastasizes. The incidence rate is reported to be ~5 per 1 million persons annually (1). Typically, DFSP starts as a red or blue-red coloured nodules. Nodules may gradually develop to become irregularly shaped swellings (2). The five-year survival rate can reach 88.9%. DFSP is relatively resistant to chemotherapy and radiotherapy, thus surgery is the primary treatment for DFSP (3). DFSP frequently involves the trunk. While the head, neck and extremities are commonly involved in DFSP, breast involvement is rare (4). Therefore, DFSP is often misdiagnosed as a benign breast tumor, delaying treatment.

## Case report

### Patient presentation

A 26-year old woman underwent ablation of a left breast lump, which was diagnosed as DFSP one year previously at YueBei People’s Hospital, Shaoguan, China). Six months after surgery, the patient noticed a lump gradually growing underneath the scarred area of the surgical site of the left breast. Physical examination revealed a brown-red, firm, fixed breast mass with an ill-defined border and smooth margins. The patient had no history of systemic disease or malignancy and the laboratory data were normal.

### Ultrasound imaging

**Figure 1:**
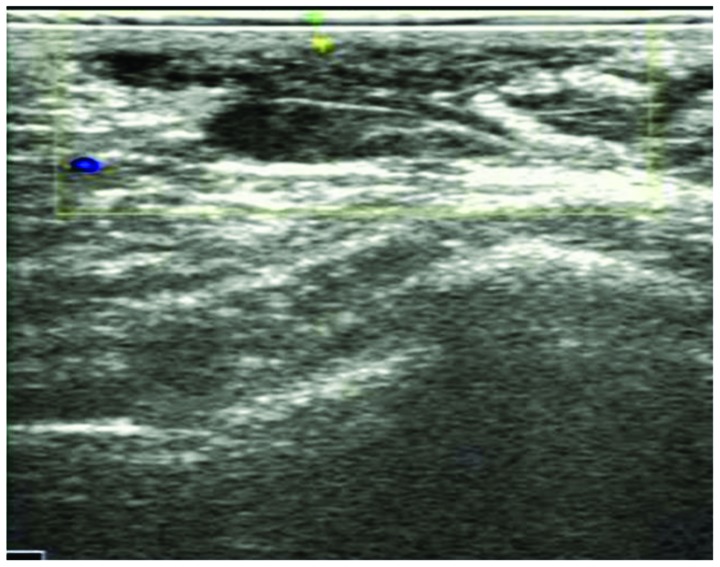
Breast ultrasonography revealing the subdermal location of the tumor, its adherence to the skin and its invasion of the subcutaneous tissue layer. No significant blood flow was observed around or within the tumor.

Targeted ultrasound of the left breast revealed a 32×9-mm hypoechoic mass lesion with an irregular border at the five o’clock position, 2 mm deep in the skin and ~40 mm from the nipple. No peripheral or internal blood flow was observed. Local invasion of subcutaneous fat and skin was also detected. The mass was classified as a Breast Imaging-Reporting and Data System-ultrasonography 4 lesion (Fig. 1).

### Surgery and follow-up

**Figure 2:**
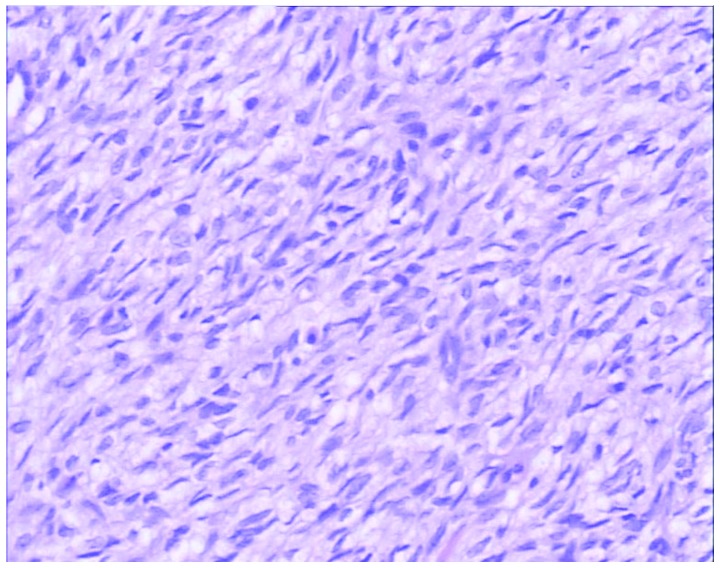
Dermatofibrosarcoma protuberans is composed of single spindle cells and forms a typical mat-like pattern.

**Figure 3:**
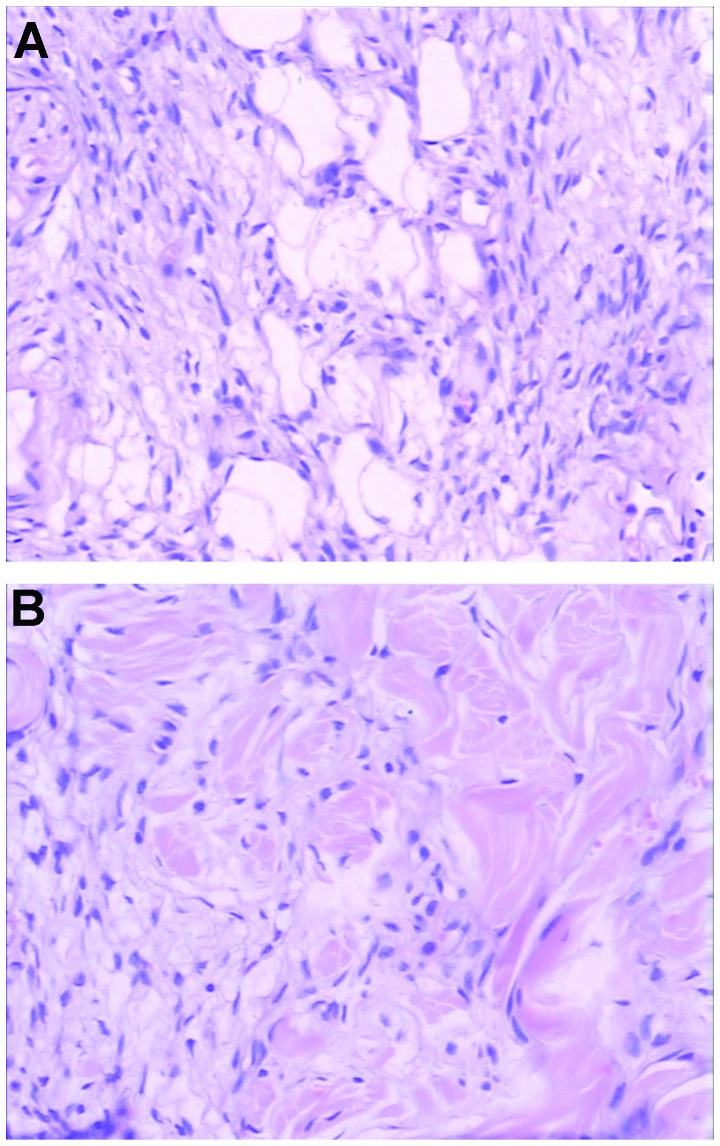

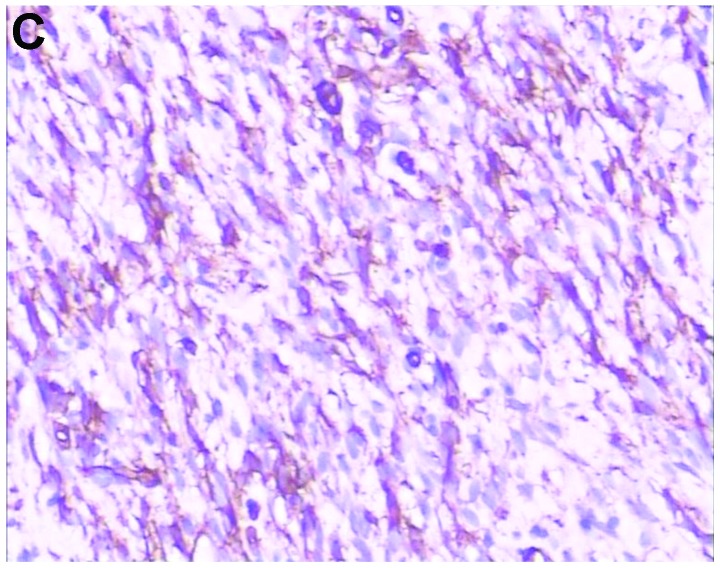
Immunohistochemistry shows that dermatofibrosarcoma protuberans is CD34-positive and invades the subcutaneous connective and adipose tissue. (A) Tumor invasion of the adipose tissue. (B) Tumor invasion of the connective tissue. (C) CD34-positive staining. CD34, cluster of differentiation 34.

The mass was excised with 3-cm margins. The patient underwent excisional breast biopsy following surgery. Pathological analysis revealed that the mass consisted of spindle cells arranged in storiform patterns and short fascicles (Fig. 2) that were cluster of differentiation (CD) 34-positive (Fig. 3). The patient was followed-up for ~12 months and did not exhibit any signs of recurrence.

## Discussion

DFSP is a rare cutaneous malignancy that arises from the dermis. The reported incidence of DFSP is approximately five cases per one million individuals per year (5). DFSP was first described by Darier and Ferrand (6) in 1924, and was termed DFSP by Hoffmann (7) in 1925. While DFSP is a low-grade sarcoma, it is capable of infiltration and local recurrence following inadequate excision.

DFSP usually involves the trunk. DFSP is also often reported in the limbs, head and neck; however, involvement of the breast is rare, as described previously (8). Typically, DFSP develops as a deep-red or blue-red plaque and grows slowly, usually reaching a size of ≥3 cm (2).

DFSP is a relatively rare cancer of the breast and is difficult to diagnose. DFSP may be difficult to distinguish from mammary fibroadenomas due to the lack of peripheral or internal blood flow and the oval or spherical lump observed using B-mode ultrasound. However, the high rate of local spreading and the involved area of the dermis differentiate DFSP from other mammary fibroadenomas (1,9).

Surgery is the preferred treatment option for DFSP. Due to the high recurrence rate associated with DFSP, treatment consists of radical excision, which involves either complete surgical excision with wide margins (>3 cm) performed during conventional surgery or Mohs micrographic surgery. Selective or superselective lymphadenectomy is not important. As in the present case, pathological analysis reveals spindle cells arranged in storiform patterns and short fascicles (2,10) that are CD34-positive (3,10,11). Postoperative recurrence has been associated with the index of cell division, cell structure, and incisal margin (12). In the present case, the patient was advised to undergo a physical examination and breast B-mode ultrasound examination twice a year in the first year, followed by annual physical and B-mode ultrasound examinations.

Due to the rare involvement of the breast in cases of DFSP, the present study reports this unique case with the clinical features and B-mode ultrasound imaging findings. Diagnostic imaging examinations are useful tools for pre-surgical examination of breast DFSP, as well as for detecting its post-surgical recurrence.
